# Machine learning-based prognostic model for triple-negative breast cancer with axillary lymph node metastasis

**DOI:** 10.3389/fonc.2026.1874031

**Published:** 2026-07-10

**Authors:** Ruyi Huang, Tianlu Jiang, Xidong Lv, Na Yao, Yujiang Guo

**Affiliations:** Department of Breast Surgery, The Affiliated Wuxi People’s Hospital of Nanjing Medical University, Wuxi People’s Hospital, Wuxi Medical Center, Nanjing Medical University, Wuxi, Jiangsu, China

**Keywords:** axillary lymph node metastasis, precision oncology, prognostic model, survival machine learning, triple-negative breast cancer (TNBC)

## Abstract

**Background:**

Triple-negative breast cancer (TNBC) with axillary lymph node metastasis (ALNM) represents a high-risk population with substantially worse prognosis compared to other breast cancer subtypes. Despite the critical clinical importance of accurate prognostic assessment in this population, no validated risk prediction model specifically tailored for TNBC patients with ALNM currently exists. Machine learning approaches offer the potential to integrate multiple clinical and pathological factors for improved risk stratification, yet their application in this specific context remains unexplored.

**Methods:**

This retrospective study analyzed 19,289 TNBC patients with ALNM from the Surveillance, Epidemiology, and End Results (SEER) database (2015-2020). Patients were randomly allocated to training (n=13,502, 70%) and validation (n=5,787, 30%) cohorts. Independent prognostic factors were identified through univariable and multivariable Cox regression analysis. Five machine learning-based survival models were developed and compared: Cox Proportional Hazards (CoxPH), Random Survival Forest (RSF), Extremely Randomized Survival Trees (ERST), Gradient Boosting Survival Analysis (GBSA), and Survival Tree (ST). Model performance was evaluated using concordance index (C-index), time-dependent area under the curve (AUC), Brier scores, calibration curves, and decision curve analysis. SHapley Additive exPlanations (SHAP) analysis was employed to enhance model interpretability and identify key prognostic drivers.

**Results:**

Multivariable Cox regression identified 13 independent prognostic factors encompassing demographic characteristics (age, race, marital status, household income), tumor pathological features (histology type, T stage, N stage, M stage, tumor grade, tumor size), and treatment modalities (surgery, radiotherapy, chemotherapy). The four ensemble/regression models (ERST, RSF, GBSA, CoxPH) demonstrated comparable C-indices (0.7494, 0.7489, 0.7483, and 0.7455, respectively) and substantially outperformed ST (0.6959); ERST was selected for downstream SHAP interpretation given its marginally highest C-index. Time-dependent AUC values for 1-, 3-, and 5-year survival predictions were 0.889, 0.773, and 0.740, respectively, with corresponding Brier scores of 0.014, 0.067, and 0.151, indicating excellent discriminatory ability and calibration. Decision curve analysis confirmed favorable clinical utility across a wide range of threshold probabilities. SHAP analysis revealed tumor grade, N stage, and radiotherapy as the three most influential prognostic factors, with high tumor grade, advanced nodal stage, and absence of radiotherapy consistently associated with increased mortality risk.

**Conclusions:**

We developed and internally validated the first machine learning-based prognostic model specifically for TNBC patients with ALNM, integrating 13 clinicopathological variables. The ERST model demonstrated robust discriminatory performance, excellent calibration, and favorable clinical utility. SHAP-based interpretability analysis provided transparent insights into key prognostic drivers, facilitating individualized risk assessment and clinical translation. This tool addresses a critical gap in precision oncology for this high-risk population and has the potential to inform treatment decisions, optimize surveillance strategies, and improve prognostic counseling. Future prospective validation in independent cohorts and integration of molecular biomarkers represent important next steps toward clinical implementation.

## 1 Introduction

Triple-negative breast cancer (TNBC)—defined by the absence of estrogen receptor (ER), progesterone receptor (PR), and human epidermal growth factor receptor 2 (HER2) expression—represents an aggressive entity accounting for 15%-20% of all BC cases (1). TNBC further displays marked molecular heterogeneity, with gene expression profiling classifying it into distinct molecular subtypes that differ in biological behaviors, disease progression trajectories, and treatment sensitivities (2). The lack of actionable intrinsic biomarkers renders endocrine therapy and conventional HER2-targeted agents ineffective (3), presenting a unique therapeutic challenge for clinicians (4). Epidemiological evidence indicates a 5-year survival rate of 77% following TNBC diagnosis, notably lower than the 91% observed in other BC subtypes (5).

Axillary lymph node metastasis (ALNM) is one of the most common metastatic sites in breast cancer (BC), with critical implications for clinical staging, treatment stratification, and prognostic assessment. It also serves as a key indication for neoadjuvant chemotherapy (6, 7). Studies have demonstrated that BC patients without ALNM achieve a 5-year survival rate of 99.0%, whereas this figure drops to 86.0% in those with ALNM (8). Accumulating evidence confirms that TNBC patients carry a heightened risk of developing ALNM (7).

Despite the well-established prognostic significance of axillary lymph node metastasis in TNBC, existing risk stratification tools remain limited, relying primarily on traditional TNM staging. To date, no validated prognostic model specifically tailored for TNBC patients with nodal involvement has been developed, leaving a critical gap in precision oncology for this high-risk population. Machine learning approaches offer the potential to integrate multiple clinical and pathological factors for improved risk prediction, yet their application in this specific context remains unexplored.

To address this gap in prognostic modeling for TNBC with ALNM, the present study analyzed a retrospective cohort from seer dataset of TNBC patients with ALNM. We compared traditional survival analysis with survival machine learning models to enhance prediction accuracy and employed SHapley Additive exPlanations (SHAP) to elucidate the importance of individual feature factors and identify prognosis-related variables in TNBC with ALNM. By integrating these approaches, our work not only improves the accuracy of TNBC survival prediction but also provides transparent insights into key prognostic factors, facilitating personalized treatment decisions and advancing precision oncology initiatives.

## 2 Materials and methods

### 2.1 Data acquisition and extraction

This retrospective study was conducted using data from the Surveillance, Epidemiology, and End Results (SEER) Program (SEER*Stat Database: Incidence - SEER Research Data, 8 Registries, Nov 2024 Sub (1975–2022); Surveillance Research Program, National Cancer Institute, released April 2025, based on the November 2024 submission). Since the present study analyzed de-identified public surveillance data, informed consent was waived by the Institutional Review Board (IRB). All procedures were performed in accordance with the ethical standards of the Declaration of Helsinki. The study dataset comprised patients diagnosed with breast cancer from January 1, 2015, to December 31, 2020, with all cases extracted from the most updated SEER database.

**Figure 1:**
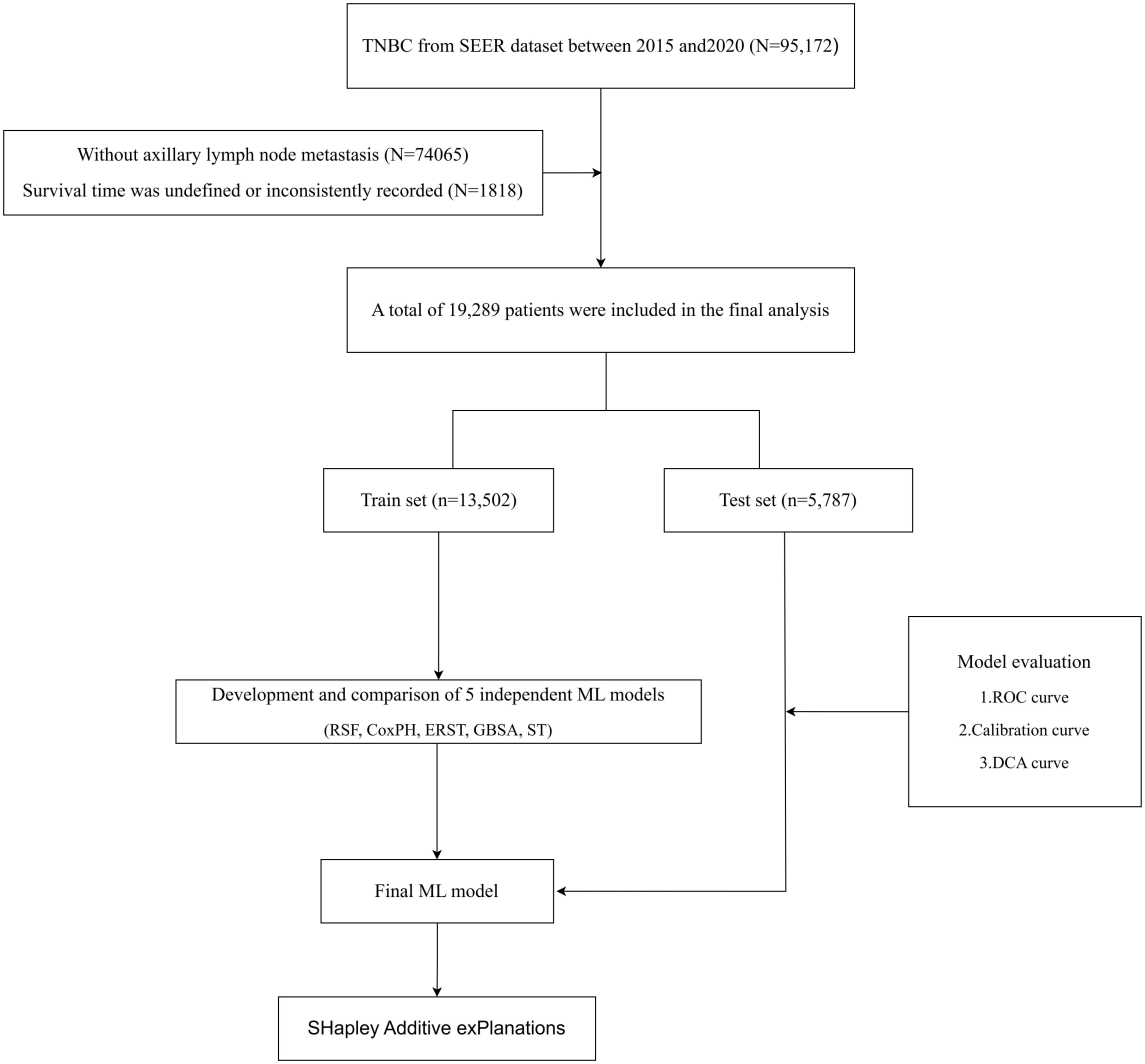
Flow chart.

A total of 95,172 patients diagnosed with breast cancer between 2015 and 2020 were initially identified from the SEER database. Patients were included if they met the following criteria: (1) histopathologically confirmed breast cancer; (2) documented presence of axillary lymph node metastasis; and (3) triple-negative phenotype confirmed by negative estrogen receptor, progesterone receptor, and HER2 expression status. Patients were excluded if survival outcome data were missing or unreliable. After applying these criteria, 19,289 patients were eligible for analysis and were randomly allocated to training (n=13,502, 70%) and internal validation (n=5,787, 30%) cohorts, as illustrated in **Figure 1**.

### 2.2 Variable extraction

A comprehensive set of risk factors was extracted for analysis, including: age at diagnosis, race (categorized as White/Not White), marital status (categorized as married and single/divorced/separated/widowed [DSW]), residential population size (<1 million inhabitants, ≥1 million inhabitants), median household monthly income (<$75,000, ≥$75,000), histological subtype (ductal carcinoma, lobular carcinoma, other), tumor grade (grade I–II, grade III), T stage, N stage, M stage, surgical intervention status, tumor size (defined as the maximum diameter of the primary tumor: ≤3 cm, >3 cm), chemotherapy and radiotherapy administration status, vital status, and overall survival time.

### 2.3 Missing data imputation

Missing data represent a prevalent challenge in clinical research. Conventional complete-case analysis (i.e., simple deletion of incomplete cases) may lead to substantial information loss and inefficient utilization of valuable clinical data. To address this issue, data imputation is a more scientific and methodologically robust approach. Multiple imputation was adopted for missing data processing in this study, a method that has been extensively validated and demonstrated superior performance in numerous clinical investigations (9, 10). The mice package in R software was used to impute variables with a missing data rate of <20%. For methodological rigor, multiple imputation was performed on the full dataset prior to the training/validation split, ensuring consistent missing-data handling. Results from Cox regression were combined across imputed datasets using Rubin’s rules; the first imputed dataset (m=1) was used for ML model training. A complete-case sensitivity analysis was additionally performed to confirm the robustness of the main findings. Thereafter, the dataset was split into a training set and an internal test set at a ratio of 7:3 to facilitate robust model development and internal validation.

### 2.4 Selection of prognostic features

To enhance model transferability and interpretability, prognostic feature selection was based on risk factor identification combined with clinical expert consensus. First, univariate Cox proportional hazards regression analysis was performed, and variables with a two-sided P-value <0.05 were included in subsequent multivariate Cox regression analysis with collinearity assessment. Variables with a two-sided P-value <0.05 in the multivariate analysis were further evaluated by clinical experts, and those confirmed as independent prognostic risk factors were finally incorporated into the predictive models.

### 2.5 Prognostic model development and validation

Based on the prognostic features screened via the above process, five machine learning-based survival models were constructed to predict the prognosis of TNBC patients with axillary lymph node metastasis, with the aim of guiding clinical decision-making: Cox proportional hazards model (CoxPH), Random Survival Forest (RSF), Survival Tree (ST), Extremely Randomized Survival Trees (ERST), and Gradient Boosting Survival Analysis (GBSA). The concordance index (C-index) was used to select the optimal model among the five candidates. The optimal model was evaluated across three key dimensions: discrimination (assessed using C-index and time-dependent Area Under the Curve [AUC] (11)), calibration (evaluated via calibration curves and integrated Brier score, where C-index measures the model’s ability to distinguish between patients who experience events and those who do not, and Brier score quantifies the calibration and goodness-of-fit of predicted survival probabilities (12)), and clinical utility (assessed using Decision Curve Analysis [DCA]). To ensure comparability across algorithms, all ML models were trained using default hyperparameter settings, thereby avoiding performance bias introduced by algorithm-specific tuning. To rigorously assess model robustness, 1000 times of bootstrap resampling were performed in addition to the initial 7:3 dataset splitting. The average C-index across all bootstrap iterations was calculated to correct for over-optimism bias. Risk stratification was conducted based on the optimal model, and Kaplan-Meier (K-M) survival analysis with the log-rank test was used to compare survival differences among different risk subgroups.

### 2.6 Model interpretation

SHapley Additive exPlanations (SHAP) is a game theory-based approach designed to interpret black-box models such as machine learning systems (13). By providing both instance-specific local interpretations and model-wide global interpretations, the SHAP method quantifies the contribution of each feature to the predicted outcome, thereby improving model transparency and interpretability. In this study, the optimal model selected via multi-model comparison was used as the predictive framework, and the prognostic risk factors were systematically ranked in descending order based on their feature importance scores derived from the SHAP method.

### 2.7 Statistical analysis

All data analyses and model development were performed using R software (version 4.4.0) and Python (version 3.11). Continuous variables following a normal distribution are presented as “mean ± standard deviation”, while non-normally distributed continuous variables are expressed as “median (interquartile range, Q1-Q3)”. Group comparisons for normally distributed variables were conducted using independent samples t-test, and non-parametric tests (Mann-Whitney U test or Kruskal-Wallis test) were used for non-normally distributed variables. Categorical variables are presented as “n (%)”, with group comparisons performed using chi-square test or Fisher’s exact test as appropriate. Survival curves were generated using the Kaplan-Meier method, and differences between curves were compared using log-rank test. Hazard ratios (HRs) were calculated using the Cox proportional hazards model. All tests were two-tailed, with statistical significance defined as P < 0.05. 

## 3 Results

### 3.1 Patient characteristics

A total of 19,289 patients with TNBC and ALNM from the SEER database were ultimately included in this study, comprising 13,502 patients in the training cohort and 5,787 patients in the validation cohort. The baseline demographic and clinicopathological characteristics of the two cohorts are summarized in **Table 1**. No statistically significant differences were observed between the training and validation cohorts for any variable (all P>0.05), indicating good comparability between the two groups and supporting the appropriateness of the dataset partition for subsequent model development and validation.

**Table 1:** Demographic and clinical characteristics of patients of TNBC with ALNM.

Variables	Train cohort	Validation cohort	t/χ2	p
Age			0.164	0.686
<50	4174 (30.91)	1772 (30.62)		
>=50	9328 (69.09)	4015 (69.38)		
Race			0.052	0.819
Other	3425 (25.37)	1477 (25.52)		
White	10077 (74.63)	4310 (74.48)		
Marital status			0.907	0.341
Married	8425 (62.4)	3569 (61.67)		
Other	5077 (37.6)	2218 (38.33)		
Household income			1.496	0.221
<75000	2622 (19.42)	1080 (18.66)		
≥75000	10880 (80.58)	4707 (81.34)		
Habitat			0.636	0.425
< 1 million pop	5684 (42.1)	2472 (42.72)		
> 1 million pop	7818 (57.9)	3315 (57.28)		
Histology type			0.784	0.676
Ductal Carcinoma	10528 (77.97)	4533 (78.33)		
Lobular Carcinoma	2715 (20.11)	1153 (19.92)		
Other/Unknown	259 (1.92)	101 (1.75)		
T stage			1.998	0.573
T1	4130 (30.59)	1732 (29.93)		
T2	7335 (54.33)	3140 (54.26)		
T3	1990 (14.74)	893 (15.43)		
T4	47 (0.35)	22 (0.38)		
N stage			2.261	0.323
N1	11254 (83.35)	4798 (82.91)		
N2	1051 (7.78)	438 (7.57)		
N3	1197 (8.87)	551 (9.52)		
M stage			1.901	0.168
M0	13091 (96.96)	5632 (97.32)		
M1	411 (3.04)	155 (2.68)		
Grade			2.102	0.147
I/II	8872 (65.71)	3865 (66.79)		
III	4630 (34.29)	1922 (33.21)		
Tumor_size			0.185	0.667
> 30 mm	4995 (36.99)	2122 (36.67)		
≤ 30 mm	8507 (63.01)	3665 (63.33)		
Surgery			0.557	0.906
Breast-Conserving Surgery	6111 (45.26)	2634 (45.52)		
Mastectomy	3951 (29.26)	1688 (29.17)		
No	3324 (24.62)	1410 (24.36)		
Unknown	116 (0.86)	55 (0.95)		
Radiotherapy			1.215	0.27
None/Unknown	3089 (22.88)	1282 (22.15)		
Yes	10413 (77.12)	4505 (77.85)		
Chemotherapy			1.23	0.267
No/Unknown	4133 (30.61)	1818 (31.42)		
Yes	9369 (69.39)	3969 (68.58)		
Status			0.059	0.808
alive	11702 (86.67)	5023 (86.8)		
dead	1800 (13.33)	764 (13.2)		
Time (months)	54.51 ± 22.36	54.32 ± 22.43	0.533	0.594

With respect to demographic characteristics, most patients were aged ≥50 years, accounting for 69.2% of the training cohort and 69.1% of the validation cohort. White patients constituted the majority in both cohorts (74.91% vs. 73.84%). Most patients were married (61.99% vs. 62.62%), had a household income ≥$75,000 (80.68% vs. 81.11%), and lived in metropolitan areas with a population >1 million (57.44% vs. 58.37%).

Regarding clinicopathological characteristics, ductal carcinoma was the predominant histological subtype (78.34% in the training cohort and 77.48% in the validation cohort). T2 stage was the most common T stage (54.41% vs. 54.05%), while N1 stage was the predominant nodal stage (83.37% vs. 82.88%). The vast majority of patients were classified as M0 stage (97.06% vs. 97.08%). Most tumors were grade I/II (66.06% in the training cohort and 65.98% in the validation cohort), and tumor size ≤30 mm was observed in 63.26% and 62.73% of patients, respectively.

In terms of treatment, breast-conserving surgery was the most frequently performed surgical procedure (45.54% vs. 44.86%), followed by mastectomy (29.17% vs. 29.38%). Radiotherapy was administered in 77.28% of patients in the training cohort and 77.48% in the validation cohort, while chemotherapy was received by 69.10% and 69.26% of patients, respectively.

During follow-up, the mean follow-up time was 54.40 ± 22.29 months in the training cohort and 54.56 ± 22.58 months in the validation cohort. At the last follow-up, 86.9% of patients in the training cohort were alive and 13.1% had died, compared with 86.26% alive and 13.74% deceased in the validation cohort. Overall, no statistically significant differences were identified between the two cohorts across all baseline variables, indicating that the training and validation cohorts were well balanced.

### 3.2 Feature selection

To identify prognostic factors associated with overall survival in TNBC patients with ALNM, Cox proportional hazards regression analyses were performed using clinicopathological variables. First, univariable Cox regression analysis was conducted to screen variables significantly associated with overall survival. Variables with P<0.05 in the univariable analysis were subsequently entered into the multivariable Cox regression model to determine independent prognostic factors.

In the univariable Cox regression analysis, all variables except surgery category “mastectomy” were significantly associated with overall survival (**Table 2**). Specifically, age, race, marital status, household income, habitat, histology type, T stage, N stage, M stage, grade, tumor size, most surgery categories, radiotherapy, and chemotherapy were all significantly associated with prognosis in univariable analysis.

**Table 2:** Univariable and multivariable Cox regression analysis for overall survival in TNBC patients with ALNM.

Variables	Category	Univariable HR (95% CI), P value	Multivariable HR (95% CI), P value
Age	<50		
	>=50	1.47 (1.34-1.61, p<0.001)	1.52 (1.38-1.67, p<0.001)
Race	Other		
	White	0.81 (0.74-0.88, p<0.001)	0.90 (0.83-0.99, p=0.028)
Marital status	Married		
	Other	1.70 (1.57-1.83, p<0.001)	1.49 (1.38-1.62, p<0.001)
Household income	<75000		
	>75000	0.75 (0.69-0.82, p<0.001)	0.83 (0.75-0.92, p<0.001)
Habitat	< 1 million pop		
	> 1 million pop	0.90 (0.83-0.97, p=0.006)	0.95 (0.87-1.04, p=0.238)
Histology type	Ductal Carcinoma		
	Lobular Carcinoma	0.84 (0.76-0.93, p=0.001)	0.86 (0.77-0.96, p=0.006)
	Other/Unknown	1.81 (1.45-2.25, p<0.001)	1.51 (1.21-1.88, p<0.001)
T stage	T1		
	T2	2.00 (1.79-2.24, p<0.001)	1.39 (1.23-1.57, p<0.001)
	T3	3.71 (3.28-4.20, p<0.001)	1.82 (1.54-2.14, p<0.001)
	T4	7.40 (5.24-10.45, p<0.001)	2.55 (1.77-3.67, p<0.001)
N stage	N1		
	N2	1.92 (1.72-2.15, p<0.001)	1.55 (1.38-1.75, p<0.001)
	N3	3.47 (3.15-3.83, p<0.001)	2.42 (2.17-2.69, p<0.001)
M stage	M0		
	M1	5.03 (4.42-5.71, p<0.001)	2.38 (2.07-2.72, p<0.001)
Grade	I/II		
	III	2.44 (2.25-2.63, p<0.001)	2.08 (1.91-2.26, p<0.001)
Tumor size	> 30 mm		
	≤ 30 mm	0.43 (0.40-0.47, p<0.001)	0.69 (0.62-0.77, p<0.001)
Surgery	Breast-Conserving Surgery		
	Mastectomy	1.07 (0.96-1.18, p=0.220)	0.84 (0.75-0.93, p=0.001)
	No	2.13 (1.95-2.33, p<0.001)	1.11 (1.01-1.23, p=0.039)
	Unknown	1.93 (1.38-2.69, p<0.001)	1.14 (0.81-1.60, p=0.443)
Radiotherapy	None/Unknown		
	Yes	0.48 (0.44-0.52, p<0.001)	0.52 (0.48-0.57, p<0.001)
Chemotherapy	No/Unknown		
	Yes	1.18 (1.08-1.28, p<0.001)	0.81 (0.74-0.90, p<0.001)

Multivariable Cox regression analysis demonstrated that several variables remained independent prognostic factors for overall survival. Adverse prognostic factors included older age (≥50 years: HR = 1.52, 95% CI: 1.38-1.67, P<0.001), unmarried status (HR = 1.49, 95% CI: 1.38-1.62, P<0.001), advanced T stage (T2: HR = 1.39; T3: HR = 1.82; T4: HR = 2.55; all P<0.001), higher nodal burden (N2: HR = 1.55; N3: HR = 2.42; both P<0.001), M1 stage (HR = 2.38, 95% CI: 2.07-2.72, P<0.001), grade III tumors (HR = 2.08, 95% CI: 1.91-2.26, P<0.001), and absence of surgery compared with breast-conserving surgery (HR = 1.11, 95% CI: 1.01-1.23, P = 0.039). In addition, patients with other/unknown histology had worse survival than those with ductal carcinoma (HR = 1.51, 95% CI: 1.21-1.88, P<0.001).

Several factors were identified as protective. White race was associated with a lower risk of death compared with other races (HR = 0.90, 95% CI: 0.83-0.99, P = 0.028). Higher household income (>$75,000) was associated with better survival (HR = 0.83, 95% CI: 0.75-0.92, P<0.001). Compared with ductal carcinoma, lobular carcinoma was associated with a more favorable prognosis (HR = 0.86, 95% CI: 0.77-0.96, P = 0.006). Mastectomy was associated with improved survival compared with breast-conserving surgery (HR = 0.84, 95% CI: 0.75–0.93, P = 0.001). Tumor size ≤30 mm was also a protective factor (HR = 0.69, 95% CI: 0.62-0.77, P<0.001). Furthermore, radiotherapy (HR = 0.52, 95% CI: 0.48-0.57, P<0.001) and chemotherapy (HR = 0.81, 95% CI: 0.74-0.90, P<0.001) were both independently associated with improved overall survival.

Notably, although habitat was significant in the univariable analysis (HR = 0.90, 95% CI: 0.83-0.97, P = 0.006), it did not retain statistical significance in the multivariable model (HR = 0.95, 95% CI: 0.87-1.04, P = 0.238).Notably, mastectomy showed a non-significant association in univariable analysis (HR = 1.07, P = 0.220) but emerged as a protective factor in multivariable analysis (HR = 0.84, P = 0.001). This sign reversal is likely attributable to the confounding effects of tumor stage and size: after adjusting for these variables, the true protective effect of mastectomy becomes apparent, as it is preferentially employed in patients with more extensive locoregional disease. Based on the results of Cox regression analysis and clinical relevance, 13 clinicopathological variables, including age, race, marital status, household income, histology type, T stage, N stage, M stage, grade, tumor size, surgery, radiotherapy, and chemotherapy, were selected for subsequent machine learning model construction.

### 3.3 Model performance

Based on the thirteen, selected prognostic variables, we constructed and compared the performance of five survival analysis machine learning models, including Random Survival Forest (RSF), Cox Proportional Hazards (CoxPH), Extremely Randomized Survival Trees (ERST), Gradient Boosting Survival Analysis (GBSA), and Survival Tree (ST). Model performance was evaluated using the Concordance Index (C-index), with values closer to 1 indicating superior predictive performance.

**Table 3** presents the predictive performance of the five models in the validation cohort. The results demonstrated that the four ensemble/regression models (ERST: 0.7494, RSF: 0.7489, GBSA: 0.7483, CoxPH: 0.7455) achieved comparable C-indices, with a maximum spread of only 0.0039, indicating performance differences within the expected range of statistical noise. All four models substantially outperformed the single Survival Tree (ST: 0.6959). Given this overall equivalence among the top four models, we selected ERST as the downstream interpretive model based on its marginally highest C-index and its favorable compatibility with SHAP-based interpretability frameworks.

**Table 3:** Performance metrics of five survival machine learning models.

Model	RSF	CoxPH	ERST	GBSA	ST
C-index	0.7489	0.7455	0.7494	0.7483	0.6959

C-index, Concordance Index; RSF, Random Survival Forest; CoxPH, Cox Proportional Hazards; ERST, Extremely Randomized Survival Trees; GBSA, Gradient Boosting Survival Analysis; ST, Survival Tree.

Model performance based on the 13 variables is presented in **Table 3**. The ERST model achieved the highest C-index (0.7494) and was therefore selected as the optimal predictive model.

**Figure 2:**
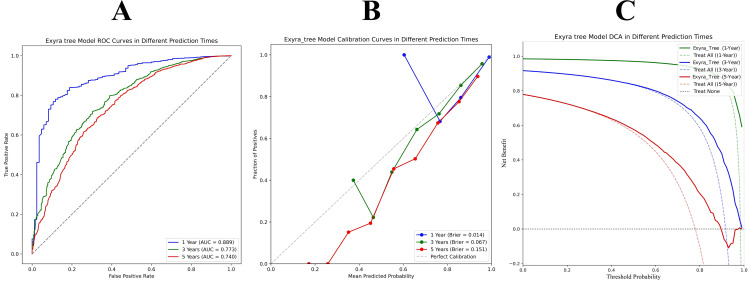
Comprehensive evaluation of Extremely Randomized Survival Trees model performance. **(A)** time-dependent ROC curves, **(B)** Calibration curve, **(C)** decision curve analysis.

To further evaluate the predictive accuracy and clinical utility of the ERST model, we calculated time-dependent area under the receiver operating characteristic curve (AUC) and Brier scores for 1-, 3-, and 5-year survival predictions. As illustrated in **Figure 2**, the ERST model achieved AUC values of 0.889, 0.773, and 0.740 for 1-, 3-, and 5-year predictions, respectively, indicating excellent discriminatory ability for both short-term and intermediate-to-long-term survival predictions. The corresponding Brier scores were 0.014, 0.067, and 0.151, with values closer to 0 indicating lower prediction error, further confirming the high predictive accuracy of the model. Decision curve analysis (DCA) demonstrated that the ERST model exhibited positive net benefit across a wide range of threshold probabilities, establishing its favorable practical value for clinical decision-making.

### 3.4 Model interpretation

To enhance model interpretability and facilitate clinical translation, we employed SHapley Additive exPlanations (SHAP) to elucidate the contribution of each prognostic variable to model predictions. SHAP values provide a unified measure of feature importance and enable visualization of how individual features influence specific predictions, thereby bridging the gap between model complexity and clinical comprehension.

**Figure 3:**
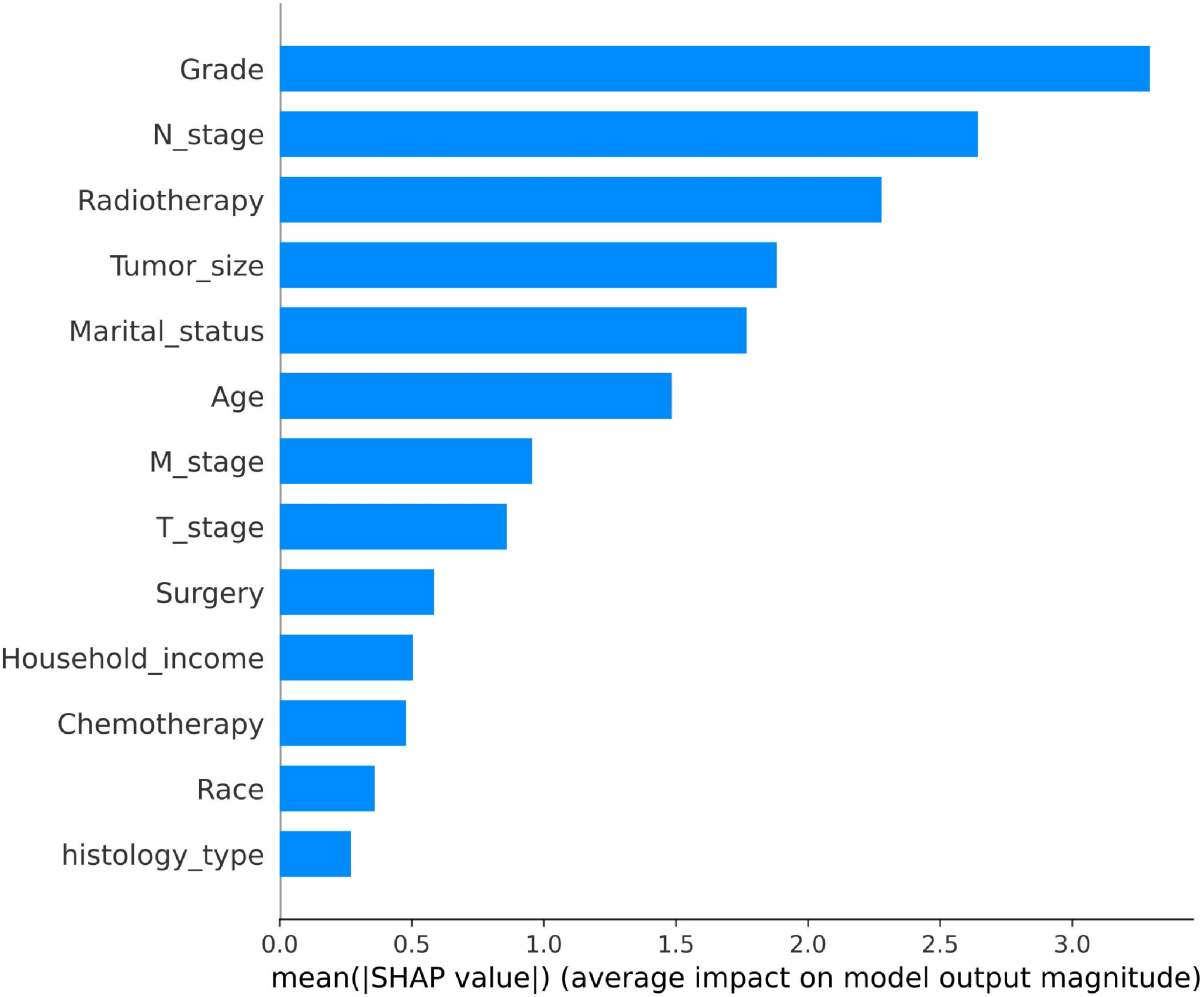
Mean absolute SHAP values of the 13 prognostic variables in the ERST model.

**Figure 3** presents the mean absolute SHAP values for all 13 prognostic variables, ranking their overall importance in the ERST model. The analysis revealed that tumor grade emerged as the most influential predictor, with the highest mean SHAP value, substantially exceeding all other variables. This finding underscores the critical prognostic significance of tumor differentiation status in TNBC with ALNM. N stage ranked second in importance, highlighting the substantial prognostic value of regional lymph node involvement extent. Radiotherapy demonstrated the third highest importance, reflecting its considerable protective effect on patient survival, consistent with the multivariable Cox regression results. Tumor size and marital status ranked fourth and fifth, respectively, indicating their considerable contribution to prognostic prediction. Age ranked sixth, followed by M stage, T stage, and surgery type. Household income, chemotherapy, race, and histology type demonstrated progressively diminishing importance, though all remained integral components of the comprehensive prognostic model.

**Figure 4:**
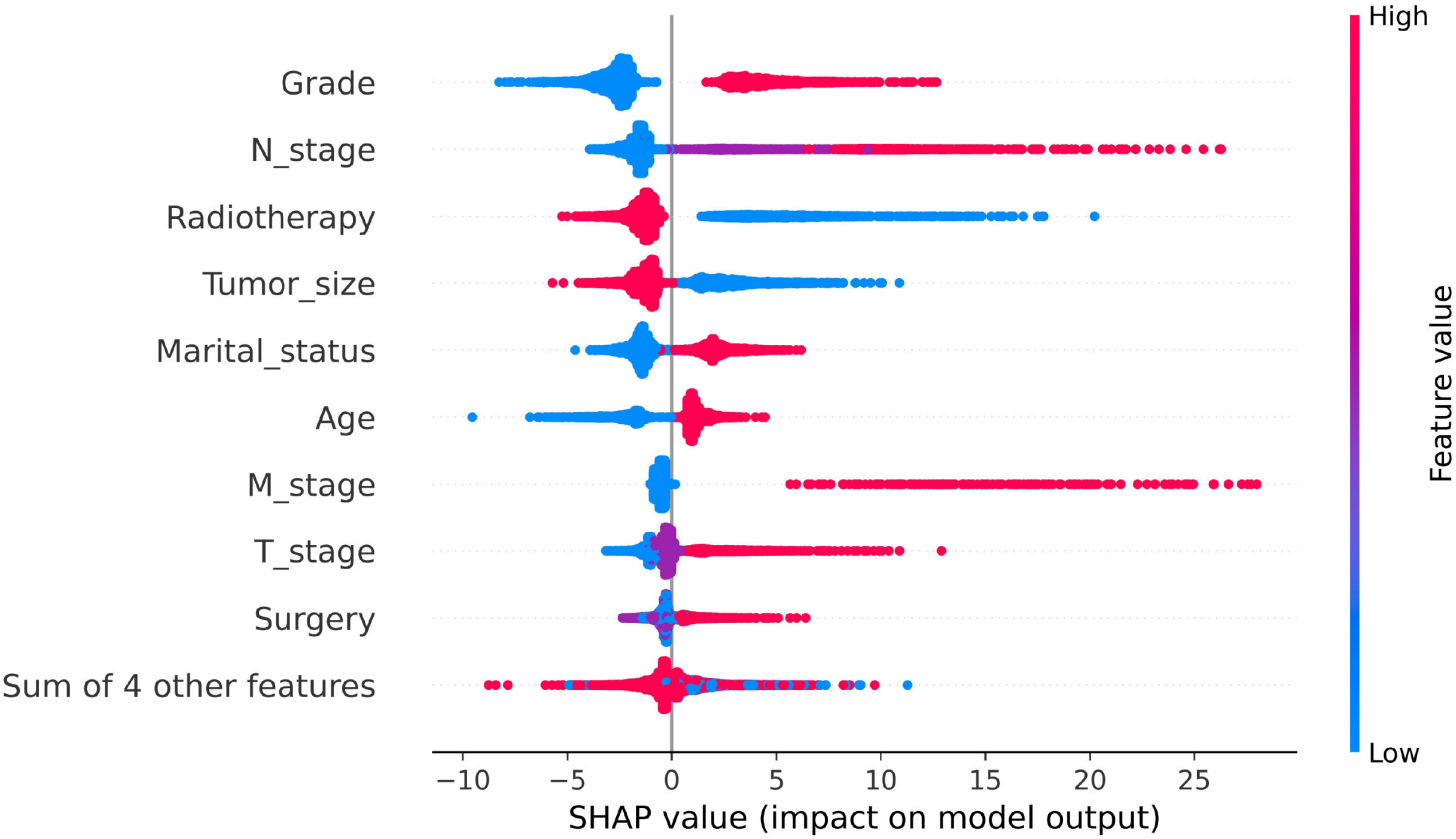
SHAP beeswarm plot showing the direction and magnitude of each feature’s impact on model output. Red indicates high feature values and blue indicates low feature values.

**Figure 4** presents the SHAP summary plot, illustrating both the magnitude and direction of each feature’s impact on model output. Each point represents an individual patient, with color indicating feature value (red = high, blue = low) and position on the x-axis representing SHAP value (impact on prediction). For tumor grade, high-grade tumors (Grade III, depicted in red) consistently exhibited positive SHAP values, indicating increased mortality risk, while low-grade tumors (Grade I/II, depicted in blue) showed negative SHAP values, conferring protective effects. This bidirectional pattern aligns with established clinical knowledge that poorly differentiated tumors portend worse prognosis. N stage exhibited a pronounced gradient effect, with higher nodal involvement stages (red) associated with strongly positive SHAP values extending to approximately +25, while lower stages (blue) conferred negative values, reflecting the progressive detrimental impact of increasing nodal burden. Radiotherapy demonstrated an inverse relationship: patients receiving radiotherapy (red points) predominantly clustered at negative SHAP values, confirming its protective role in reducing mortality risk. For tumor size, larger tumors (>30 mm, depicted in red) showed positive SHAP values indicative of elevated risk, while smaller tumors (≤30 mm, depicted in blue) demonstrated negative SHAP values. Marital status showed that married category tended toward negative SHAP values (protective), while the other shifted toward positive SHAP values (increased risk). For M stage, M1 disease (red) strongly shifted predictions toward higher risk (positive SHAP values), while M0 disease (blue) conferred lower risk (negative SHAP values). The four remaining variables—household income, chemotherapy, race, and histology type—are collectively represented as “Sum of 4 other features” in the plot, reflecting their comparatively modest individual contributions to model output.

**Figure 5:**
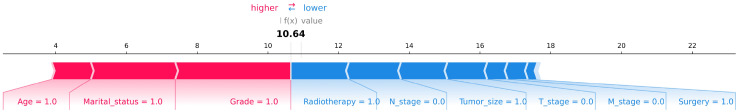
SHAP force plot for a representative individual patient (f(x) = 10.64). Pink features increase predicted risk; blue features decrease it. Marital_status = 1.0, married; Grade = 1.0, Grade III.

**Figure 5** presents a SHAP force plot for an individual patient case, demonstrating how the model integrates multiple features to generate a specific prediction. The f(x) value of 10.64 represents the model output for this particular patient. Red arrows pointing leftward (toward “higher”) indicate features that increase the predicted risk above the baseline, while blue arrows pointing rightward (toward “lower”) represent features that decrease risk. In this illustrative case, the patient’s higher age (Age=1.0), unmarried status (Marital_status=1.0), and high tumor grade (Grade=1.0) collectively pushed the prediction toward higher risk (red arrows). Conversely, receipt of radiotherapy (Radiotherapy=1.0), lower N stage (N_stage=0.0), smaller tumor size (Tumor_size=1.0), lower T stage (T_stage=0.0), absence of distant metastasis (M_stage=0.0), and surgical intervention (Surgery=1.0) exerted protective effects, pulling the prediction toward lower risk (blue arrows). This patient-level visualization facilitates individualized risk assessment and enables clinicians to identify modifiable risk factors amenable to therapeutic intervention.

In summary, this study successfully developed a comprehensive machine learning-based prognostic prediction model for TNBC patients with ALNM by incorporating 13 clinicopathological variables identified through rigorous multivariable Cox regression analysis. Through systematic model comparison and performance evaluation, the ERST model was established as the optimal predictive tool, demonstrating robust discriminatory ability (C-index=0.7494) and excellent calibration across multiple time points. SHAP-based interpretability analysis revealed that tumor grade, N stage, and radiotherapy constitute the three most influential prognostic determinants, while also elucidating the complex interplay among demographic, pathological, and treatment-related factors in shaping patient outcomes. The model’s strong performance, coupled with its transparent interpretability, positions it as a valuable clinical decision support tool for individualized risk stratification, treatment planning, and prognostic counseling in TNBC patients with axillary lymph node metastasis. Future prospective validation studies are warranted to further assess its clinical utility and generalizability across diverse patient populations and healthcare settings.

## 4 Discussion

This study developed and internally validated the first machine learning-based prognostic model specifically for TNBC patients with ALNM, addressing a critical gap in precision oncology for this high-risk population. By integrating 13 clinicopathological variables and employing SHAP-based interpretability, our model not only achieved robust discriminatory performance (C-index=0.7494) but also provided transparent insights into key prognostic drivers, facilitating clinical translation.

This comprehensive ML-based prognostic model offers several critical clinical advantages. First, by incorporating 13 diverse variables spanning demographic characteristics (age, race, marital status, household income), tumor pathology (histology type, T stage, N stage, M stage, grade, tumor size), and treatment modalities (surgery, radiotherapy, chemotherapy), the model enables clinicians to accurately predict mortality risk through a holistic assessment of patient profiles. Second, the model facilitates early identification and targeted management of high-risk individuals, allowing for intensification of surveillance or therapeutic strategies in patients predicted to have poor outcomes. Third, the SHAP-based interpretability framework enhances clinical transparency and trust, enabling the model to serve as a valuable communication tool for discussing prognosis and treatment options with patients and their families. Fourth, the identification of modifiable risk factors, particularly the protective effects of radiotherapy and chemotherapy, provides actionable insights for optimizing treatment selection.

Regarding clinical translatability, the 13 model variables can be broadly divided into two categories. The first comprises variables routinely available in standard clinical practice: age, T stage, N stage, M stage, tumor grade, tumor size, histology type, surgical approach, radiotherapy, and chemotherapy status—all of which are universally recorded in oncology documentation. The second category consists of sociodemographic variables requiring supplementary data collection, namely race, marital status and household income, which are not systematically captured in standardized clinical records outside the US registry context. For real-world implementation, these latter variables could be collected via brief structured questionnaires at the point of care, and their collection would be particularly important for applications in non-US healthcare settings.

The prognosis of TNBC patients with ALNM is highly variable, ranging from short-term mortality to long-term disease-free survival, underscoring the critical importance of identifying comprehensive prognostic predictors and developing robust risk stratification models. Our multivariable Cox regression analysis identified 13 independent prognostic factors, and these findings align with and extend existing literature across multiple domains.

Tumor grade emerged as the most influential predictor in our SHAP analysis, consistent with established evidence that histological differentiation status fundamentally reflects tumor biology and aggressiveness. Thike et al. (14) demonstrated that high histological grade constitutes an independent adverse prognostic factor in TNBC, likely attributable to altered cellular biology conferring increased malignancy and treatment resistance. The predominance of grade in our model underscores its central role in determining clinical outcomes and supports its continued emphasis in prognostic assessment and treatment stratification.

N stage demonstrated the second highest importance in our model, reflecting the well-established prognostic significance of regional lymph node involvement extent. Previous studies have established a causal relationship between lymph node burden and TNBC prognosis, with increasing numbers of metastatic nodes correlating with progressively worse outcomes (15). TNBC patients with extensive nodal involvement exhibit more aggressive clinical behavior and higher propensity for distant metastasis (16). Our finding that N3 stage independently predicted mortality (HR = 2.42, *p<*0.001) reinforces the need for comprehensive nodal staging and consideration of escalated systemic therapy in patients with high nodal burden.

Radiotherapy ranked as the third most important predictor, exhibiting substantial protective effects (HR = 0.52, *p* < 0.001). This finding aligns with accumulating evidence supporting the survival benefit of radiotherapy in TNBC. Haque et al. (17) reported that postmastectomy radiotherapy significantly improved 5-year overall survival in pT3N0M0 TNBC patients (74.3% vs. 62.6%, *p* < 0.001). Kim et al. (18) recently reviewed molecular insights supporting radiotherapy’s efficacy in TNBC, highlighting mechanisms including immunogenic cell death induction and tumor microenvironment modulation. Our results provide population-level validation of radiotherapy’s critical role in TNBC with ALNM and emphasize the importance of ensuring appropriate radiotherapy delivery in this high-risk population.

The prognostic significance of T stage and tumor size in our model corroborates established evidence that primary tumor characteristics independently influence outcomes. Advanced T stage (T4 vs. T1: HR = 2.55, *p* < 0.001) and larger tumor size (>30mm) were associated with increased mortality risk, likely reflecting greater tumor burden, enhanced metastatic potential, and more extensive locoregional disease requiring aggressive multimodal therapy (14).

M stage, despite exhibiting a high hazard ratio in multivariable analysis (HR = 2.38, *p* < 0.001), demonstrated moderate importance in SHAP analysis. This apparent discrepancy likely reflects the relatively low prevalence of M1 disease in our cohort (3.08%), limiting its contribution to overall model prediction variance. Nonetheless, the presence of distant metastasis remains a critical determinant of prognosis, with metastatic TNBC patients facing substantially reduced survival despite advances in systemic therapy (5, 19).

Chemotherapy emerged as an independent protective factor (HR = 0.81, *p* < 0.001), consistent with TNBC’s well-characterized chemosensitivity and the established role of systemic chemotherapy as a cornerstone of TNBC management. The dose-dense chemotherapy regimens, such as the iddEPC protocol (dose-dense epirubicin, paclitaxel, and cyclophosphamide), have demonstrated significant reductions in recurrence and mortality in high-risk breast cancer (20, 21). Our findings reinforce the critical importance of appropriate chemotherapy administration in TNBC patients with ALNM.

Notably, our study identified several sociodemographic factors as independent prognostic indicators, extending beyond traditional clinicopathological variables. Marital status demonstrated significant prognostic value, with unmarried patients exhibiting increased mortality risk (HR = 1.49, *p* < 0.001). This association likely reflects multifaceted mechanisms including differential access to social support, treatment adherence patterns, and healthcare resource utilization. Aizer et al. (22) demonstrated in a large SEER analysis that unmarried cancer patients across multiple tumor types exhibited worse survival, attributable to later-stage diagnosis, decreased likelihood of receiving definitive treatment, and reduced treatment completion rates. Higher household income similarly conferred protective effects (HR = 0.83, *p* < 0.001), likely reflecting enhanced access to high-quality care, ability to afford optimal treatments, and reduced financial toxicity. These findings underscore the importance of addressing social determinants of health in cancer care delivery and suggest potential benefit from targeted interventions supporting vulnerable populations.

Outcomes were more favorable among White individuals than among those of other racial groups (HR = 0.90, p=0.028), consistent with documented racial disparities in breast cancer outcomes. Multiple studies have identified worse survival in Black and other minority populations with TNBC, attributed to complex interplay of biological factors (more aggressive tumor subtypes), healthcare access disparities, socioeconomic barriers, and structural inequities (23, 24). These findings highlight the urgent need for equity-focused interventions to eliminate racial disparities in TNBC outcomes. It should be noted, however, that the binary race classification (White vs. Not White) employed in this study does not adequately capture the heterogeneity among minority groups. Documented racial disparities in TNBC primarily concern Black versus White women, whereas outcomes in Asian/Pacific Islander, Hispanic, and American Indian/Alaska Native populations follow different patterns (25, 26). Future studies should employ finer-grained race/ethnicity categories to more accurately characterize these group-specific effects.

Lobular carcinoma histology exhibited a protective effect (HR = 0.86, p=0.006) compared to ductal carcinoma, although lobular TNBC represents a relatively uncommon entity. This finding may reflect distinct biological characteristics of lobular tumors, including different patterns of metastatic spread and potentially differential treatment responsiveness (27).

Interestingly, mastectomy demonstrated superior outcomes compared to breast-conserving surgery (HR = 0.84, *p* = 0.001) in multivariable analysis. This finding likely reflects appropriate case selection, with mastectomy preferentially employed in patients with more extensive locoregional disease. When coupled with appropriate adjuvant therapy, mastectomy may provide superior locoregional control in high-risk TNBC with ALNM (28). However, this observation requires careful interpretation, as treatment selection represents a complex decision influenced by tumor characteristics, patient preferences, and clinical judgment.

Age ≥50 years emerged as an adverse prognostic factor (HR = 1.52, *p* < 0.001), potentially reflecting age-related comorbidities, decreased treatment tolerance, and possible biological differences in tumor behavior (29, 30). However, chronological age represents a heterogeneous variable, and future studies incorporating geriatric assessment tools may provide more nuanced understanding of age-related prognostic effects (29).

The observed decline in time-dependent AUC from 1-year (0.889) to 3-year (0.773) and 5-year (0.740) predictions warrants discussion. Three mechanisms likely contribute to this pattern. First, the 1-year AUC benefits from a low event rate at this early time point (most patients remain alive at one year), which tends to inflate apparent discrimination. Second, as follow-up extends, the proportion of censored observations increases substantially—approximately half the cohort was followed for fewer than five years given the 2015–2020 accrual window and mean follow-up of ~54 months—which reduces the informational content available for long-term prediction. Third, baseline clinicopathological features are subject to signal attenuation over time, as their predictive relevance for survival outcomes naturally diminishes as the disease course evolves. This pattern of AUC decline with increasing prediction horizon is a well-recognized characteristic of population-based survival models and does not materially diminish the clinical utility of the model, although it underscores the importance of incorporating dynamic clinical information for long-term prognostic assessment.

A key strength of this study is the development of a comprehensive prognostic model incorporating demographic, socioeconomic, pathological, and treatment variables, offering individualized risk prediction with transparent SHAP-based interpretation to enhance clinical utility. The large sample size from the SEER database provides robust statistical power and population-level generalizability. The systematic comparison of five machine learning algorithms enabled rigorous model selection based on objective performance metrics.

Several limitations warrant consideration. First, the retrospective SEER-based design limits access to detailed treatment protocols and molecular biomarkers, which may further refine prognostic stratification. Second, external validation in independent cohorts, particularly from non-US populations, is needed to assess generalizability. Third, the study period predates widespread immunotherapy adoption in TNBC; future models should incorporate contemporary treatment regimens. Fourth, continuous variables including age, tumor size, and household income were binarized, which may sacrifice predictive signal; tree-based ensemble methods are capable of handling continuous predictors natively, and future studies should explore retaining these variables in continuous form. Fifth, the 1-year Brier score (0.014) is low partly because the event rate at this time point is low, making the absolute value less informative as an accuracy metric; Brier scores in this study serve primarily as relative benchmarks across time points rather than absolute calibration measures. Despite these limitations, our large sample size, rigorous methodology, and transparent interpretability provide a robust foundation for clinical application and future refinement.

## 5 Conclusion

We developed and internally validated a machine learning-based prognostic model specifically for TNBC patients with axillary lymph node metastasis, integrating 13 clinicopathological variables. The ERST model demonstrated robust performance (C-index=0.7494) with excellent calibration and clinical utility. SHAP analysis identified tumor grade, radiotherapy, and nodal stage as the most influential prognostic factors while enabling transparent individualized risk assessment. This tool addresses a critical gap in precision oncology for this high-risk population and has the potential to inform treatment decisions and improve patient counseling. Prospective external validation and integration of molecular biomarkers represent important next steps toward clinical implementation.

## Data Availability

The original contributions presented in the study are included in the article/**Supplementary Material**. Further inquiries can be directed to the corresponding author.
